# Preface

**DOI:** 10.1155/DTE.7.97

**Published:** 2001

**Authors:** Mamoru Suzuki

**Affiliations:** Department of Otolaryngology Tokyo Medical University Tokyo Japan


*Diagnostic and Therapeutic Endoscopy*, Vol. 7, p. 97
Reprints available directly from the publisher
Photocopying permitted by license only

© 2001 OPA (Overseas Publishers Association) N.V.
Published by license under
the Harwood Academic Publishers imprint,
part of Gordon and Breach Publishing
a member of the Taylor & Francis Group.
All rights reserved.

### Preface

MAMORU SUZUKI

*Department of Otolaryngology, Tokyo Medical University, Tokyo, Japan*

The scope of otorhinolaryngology includes the ear, nose, pharynx, larynx, upper esophagus, trachea, maxillofacial region and neck. Most of these regions are deeply located in various cavities, and hence special instruments are required for observation. Until 25 years ago, head mirrors and indirect scopes, such as otoscopes, rhinoscopes and laryngoscopes were used for examinations. The invention of the flexible fiberscope and development of the rigid endoscope enabled close observation of the ear, nose and throat. The fiberscope is most advantageous for the examination of deep and complicated structures, such as the paranasal sinuses and larynx. A specimen can even be removed via flexible forceps and a fiberscope. The invention of electronic fiberscopes which allow superfine delineation of surface structures deserves special mention. For the ear, microscopes have been more conveniently used. Recently, however, fine rigid endoscopes have been introduced even for middle ear surgery. These are particularly useful for visualizing a structure hidden behind a microscopic dead angle. Another advantage of the fiberscope and endoscope is the built-in recording system. On-the-spot recording through video systems and cameras allows quick and efficient editing of findings. Early detection of malignancies for the most part is due to the development of this electro-optical equipment. Furthermore, pain and physical stress for patients during the procedure are greatly reduced.

This special issue deals with the usefulness of the fiberscope and fine endoscope for various purposes. The usage of these scopes for the ear, nose, larynx and thyroid lesions were documented by 4 experts. Dr K. Aoki deals with the usage of a fine endoscope supplementary to surgery for middle ear cholesteatoma. Dr H. Scherer describes endonasal laser-assisted surgery. Dr S. Horiguchi presents fiberscopic findings of a laryngeal granuloma taken at different stages. Dr H. Kitano describes a novel typed of surgery for a thyroid tumor using an endoscope. All the articles give us an up-to-date perspective on otorhinolaryngological practice assisted by optical equipment. The rapid progress and great contribution of this equipment not only for diagnostic but therapeutic measures will be readily recognized.

It is the editor's pleasure if the articles in this issue are of help to readers in understanding the contribution of these optical systems in otorhinolaryngology.

